# A Replication Study for Association of *ITPKC* and *CASP3* Two-Locus Analysis in IVIG Unresponsiveness and Coronary Artery Lesion in Kawasaki Disease

**DOI:** 10.1371/journal.pone.0069685

**Published:** 2013-07-24

**Authors:** Ho-Chang Kuo, Yu-Wen Hsu, Chung-Min Wu, Shawn Hsiang-Yin Chen, Kuo-Sheng Hung, Wei-Pin Chang, Kuender D. Yang, Kai-Sheng Hsieh, Wei-Chiao Chen, Yoshihiro Onouchi, Wei-Chiao Chang

**Affiliations:** 1 Department of Pediatrics, Kaohsiung Chang Gung Memorial Hospital and Chang Gung University College of Medicine, Kaohsiung, Taiwan; 2 Department of Clinical Pharmacy, School of Pharmacy, Taipei Medical University, Taipei, Taiwan; 3 Department of Business Management, National Taipei University Technology, Taipei, Taiwan; 4 Center of Excellence for Clinical Trial and Research Graduate Institute of Injury Prevention and Control Taipei Medical University-Wan Fang Medical Center, Taipei, Taiwan; 5 Department of Healthcare Management, Yuanpei University, Hsinchu, Taiwan; 6 The Department of Medical Research, Show Chwan Memorial Hospital in Chang Bing, Changhua, Taiwan; 7 Department of Pediatrics, Kaohsiung Veterans General Hospital, Kaohsiung, Taiwan; 8 Institute of Clinical Pharmacy and Pharmaceutical Sciences, College of Medicine, National Cheng Kung University, Tainan, Taiwan; 9 Laboratory for Cardiovascular Diseases, Center for Genomic Medicine, RIKEN, Yokohama, Japan; 10 Master Program for Clinical Pharmacogenomics and Pharmacoproteomics, School of Pharmacy, Taipei Medical University, Taipei, Taiwan; 11 Department of Pharmacy, Taipei Medical University-Wanfang Hospital, Taipei, Taiwan; College of Pharmacy, University of Florida, United States of America

## Abstract

Single-nucleotide polymorphisms (SNPs) in *inositol 1,4,5-trisphosphate 3-kinase C* (*ITPKC*, rs28493229) and *caspase-3* (*CASP3*, rs113420705) are associated with susceptibility to KD in Japanese and Taiwanese populations. This study was conducted to investigate the involvement of these 2 SNPs in the risk for intravenous immunoglobulin (IVIG) resistance and coronary artery lesion (CAL) in Taiwanese population. A total of 340 KD patients were subjected to assess by the identification of 2-locus genes model. A combinatorial association between *ITPKC* (rs28493229) and *CASP3* (rs113420705) was found in CAL formation (P = 0.0227, OR: 3.06). KD patients with high-risk genotype had a trend of overrepresentation in IVIG resistance compared with individual SNPs. Our findings suggest the existence of genetic factors affecting patients’ risk for CAL formation and IVIG responsiveness in a Taiwanese population.

## Introduction

Kawasaki disease (KD) is an acute system vasculitis characterized by high fever, skin rash, redness of oral mucosa, conjunctivitis and extremities changes [1]. This disease was first described by Kawasaki et al. in 1974 [2] in English. It has been the leading cause of acquired heart diseases in children but its etiology remains unknown[3]–[5]. It occurs worldwide and mainly affects children less than 5 year-old, especially in Asia. The highest incidence was found in Japan, followed by Korea and then Taiwan[6]–[9]. The most serious complication of KD are coronary artery lesions (CAL), including myocardial infarction [10], fistula formation [11], coronary artery dilatation/ectasia and coronary artery aneurysm formation [12]. The etiology of KD is still unclear and no consistent etiologic agent for KD has been identified yet. Recently, more and more genome-wide association studies have indicated an important role of genetic polymorphisms to the susceptibility of KD [13]–[17]. Although incidence of KD varies in different ethnic population, genetic polymorphisms of *ITPKC* and *CASP3* have been shown to associate with CAL formation of KD in both Japanese and Taiwanese populations [18]–[21].

*ITPKC* gene located on chromosome 19, plays as a negative regulator of T-cell activation via Ca^2+^/NFAT signaling pathway [20]. rs28493229 is a polymorphism within intron of *ITPKC* and the C allele of rs28493229 has 8.8% minor allele frequency of KD patients in Taiwanese population [22]. rs28493229 located in the intron area has been demonstrated as a functional polymorphism for splicing efficiency [21]. *CASP3* gene located on chromosome 4 is a key molecule of cell apoptosis. Previous studies indicated a single-nucleotide polymorphism (rs113420705) located in the 5′-untranslated region of *caspase 3* (*CASP3*) [21]. The minor allele frequency of rs113420705 is 34.8% of KD patients in Taiwanese population [18]. This SNP associated with nuclear factor of activated T cell-mediated T-cell activation, is responsible for susceptibility to KD. There are likely to be several networks in the pathogenesis of KD. Both ITPKC and CASP3 involve in Ca^2+^/NFAT pathways indicating the potential role of this signaling pathway in immune system.

The efficacy of intravenous immunoglobulin (IVIG) administered in the acute phase of KD to reduce the prevalence of coronary artery abnormalities has been well-established [23]. The mechanism of action of IVIG is still elusive. IVIG appears to have a generalized anti-inflammatory effect. Possible mechanisms of IVIG include modulation of cytokine production, neutralization of bacterial super-antigens, augmentation of regulatory T activity (TGF-β), suppression of antibody synthesis inflammatory markers (CD40L, nitric oxide and iNOS expression) [24], [25] provision of anti-idiotypic antibodies, and so on [11], [26], [27]. In 2011, Shimizu et al. firstly indicated a functional role of genetic polymorphisms in TGF-β signaling pathways to the susceptibility as well as CAL formation in KD patients [28]. In the Taiwanese population, Kuo et al. also provide evidences to support a role of TGF-β signaling pathway (*TGF-β2* and *SMAD3*) in the susceptibility of KD [29].

IVIG-resistant patients are at a higher risk for CAL formation, therefore, it is important to identify an appropriate group of patient who may benefit from IVIG treatment [5], [26]. Onouchi et al. [30]. reported that both *ITPKC* and *CASP3* polymorphisms may contribute to the responsiveness of IVIG treatment response and CAL formation in KD patients [30]. This study was conducted to investigate the role of these 2 SNPs in the risk for IVIG resistance and CAL formation in a Taiwanese population.

## Materials and Methods

### Patients Studied

All subjects studied were children who fulfilled the diagnostic criteria for KD and were admitted at Chang Gung Memorial Hospital-Kaohsiung Medical Center, between 2001 and 2009. All patients were treated with a single infusion of IVIG (2 g/kg) administered over a 12-hour period. Aspirin was administered until all signs of inflammation were resolved or regression of CAL was detected under two-dimensional (2D) echocardiography as our previous studies[5], [31]–[33]. This study was approved by the Institutional Review Board of Chang Gung Memorial Hospital. All written informed consents were obtained from guardians on the behalf of the children participants involved in this study. We excluded patients who did not meet the diagnostic criteria for KD. CAL was defined by the internal diameter of the coronary artery being at least 3 mm (4 mm, if the subject was over the age of 5 years) or the internal diameter of a segment being at least 1.5 times that of an adjacent segment, as observed in the echocardiogram [18], [27], [34]. IVIG responsiveness was defined as defervescence 48 h after the completion of IVIG treatment and no fever (temperature, >38°C) recurrence for at least 7 days after IVIG with marked improvement or normalization of inflammatory signs [5], [26].

### DNA Extraction

Blood cells were subjected to DNA extraction by treating them first with 0.5% SDS lysis buffer and then protease K (1 mg/ml) for digestion of nuclear protein for 4 h at 60°C. Total DNA was harvested by using the Gentra extraction kit followed by 70% alcohol precipitation [31].

### Genotyping

Genotyping was carried out using the TaqMan Allelic Discrimination Assay (Applied Biosystems, Foster city, CA) as our previous report [18], [19], [22]. The polymerase chain reaction (PCR) was performed by using a 96-well microplate with the ABI9700 Thermal Cycler. After PCR, fluorescence was detected and analyzed using the System SDS software version 1.2.3.

### Data Analysis and Statistics

SAS 9.1 for Windows was used for analysis. Hardy-Weinberg equilibrium was assessed by the χ^2^ test with 1 degree of freedom. The statistical differences between cases and controls in genotype and allele frequency were assessed by the χ^2^-test or the Fisher exact test. The statistical differences in the genotype and allele frequency of KD patients with and those without CAL formation and patients responding to IVIG and those showing resistance were assessed using the χ^2^-test. Risk score approaches among rs28493229 and rs113420705 in *ITPKC* and *CASP3*, genes were investigated by using the Pearson’s χ^2^-test or Fisher’s exact test, gender and age were adjusted by logistic regression. The C allele of rs28493229 and the A allele of rs113420705 were considered as risk allele in KD susceptibility. KD patients with the GG genotype at one or both loci were categorized into low-risk groups, and those without the GG genotype were classified into high-risk groups as previous study in Japanese population [35].

## Results

### Basic and Clinical Characteristics of the Subjects

A total 340 KD patients were recruited in this study. Table 1 showed the characteristics of the subjects. 64.7% of patients were male. The mean age (years) and standard deviation (S.D.) were 1.7±1.7. 10.9% (37/340) of KD patients were CAL formation and 12.6% (43/340) of them are with IVIG resistance.

**Table 1:** Basal characteristics of patients with Kawasaki disease.

Characteristics	Patients with KD
	N = 340
Male gender, No. (%)	220 (64.7%)
Mean (SD) age (years)	1.7±1.7
Age range (years)	0–11
CAL formation	37 (10.9%)
IVIG resistance	43 (12.6%)

CAL: coronary artery lesions; IVIG: intravenous immunoglobulin; SD: standard deviation.

### Two-locus Association Model for Polymorphisms of *CASP3* and *ITPKC* Genes in Patients with Coronary Artery Lesion (CAL)

Although the functional SNPs of *ITPKC* and *CASP3* were identified, the combinatorial effects of these two SNPs are still unclear. Thus, we analyzed the combinatorial effects of SNPs of *ITPKC* and *CASP3* genes. As shown in Table 2, high-risk genotype group has high percentage of CAL formation than those are with low risk genotype (OR = 3.06; 95% CI = 1.12–8.33; *P* = 0.0227). However, this *P* value didn’t achieve to significance after multiple tests.

**Table 2:** Association of rs28493229 C allele and rs113420705 A allele with CAL in KD patients.

	Low-risk groupN (%)	High-risk groupN (%)	TotalN	OR (95% CI)	*P* Value
**rs28493229 G/C** a					
KD patients without CAL	266 (90.5)^b^	1 (50.0)^b^	267	9.76 (0.59–161.44)	0.1116
KD patients with CAL	28 (9.5)^b^	1 (50.0)^b^	29		
**rs113420705 G/A** a					
KD patients without CAL	239 (91.2)^c^	28 (82.4)^c^	267	2.34 (0.87–6.28)	0.0914
KD patients with CAL	23 (8.8)^c^	6 (17.6)^c^	29		
**Two-locus model**					
KD patients without CAL	246 (91.5)^d^	21 (77.8)^d^	267	3.06 (1.12–8.39)	0.0295
KD patients with CAL	23 (8.5)^d^	6 (22.2)^d^	29		

a The underlined alleles were the minor and at risk alleles. Association was evaluated under a recessive inheritance model of the risk alleles.

b KD patients with the GG or GC genotype and those with the CC genotypes were classified into low- and high-risk groups, respectively.

c KD patients with the GG or GA genotype and those with the AA genotypes were classified into low- and high-risk groups, respectively.

d KD patients with the GG genotype at one or both loci and those without the GG genotype at both loci were classified into low- and high-risk groups, respectively.

### Two-locus Association Model for Polymorphisms of *CASP3 and ITPKC* Genes in Responsiveness of Intravenous Immunoglobulin (IVIG) Treatment

We compared the allele and genotype frequencies of *ITPKC* (rs28493229) and *CASP3* (rs113420705) in IVIG responders and non-responders, respectively. As shown in the Table 3, consistent with our previous study, it was not significant between individual SNPs and IVIG resistance. However, a trend of overrepresentation of the IVIG non-responders was observed in the high-risk group by using two locus model (12.9% (low risk genotype) V.S. 18.5% (high risk genotype)).

**Table 3:** Association of rs28493229 C allele and rs113420705 A allele with IVIG response in KD patients.

	Low-risk groupN (%)	High-risk groupN (%)	TotalN	OR (95% CI)	*P* Value
**rs28493229 G/C** a					
IVIG responder	256 (86.5)^b^	2 (100.0)^b^	258	–	0.9708
IVIG non-responder	40 (13.5)^b^	0 (0.0)^b^	40		
**rs113420705 G/A** a					
IVIG responder	230 (87.1)^c^	28 (82.4)^c^	258	1.49 (0.57–3.88)	0.4155
IVIG non-responder	34 (12.9)^c^	6 (17.6)^c^	40		
**Two-locus model**					
IVIG responder	236 (87.1)^d^	22 (81.5)^d^	258	1.54 (0.54–4.33)	0.4175
IVIG non-responder	35 (12.9)^d^	5 (18.5)^d^	40		

a The underlined alleles were the minor and at risk alleles. Association was evaluated under a recessive inheritance model of the risk alleles.

b KD patients with the GG or GC genotype and those with the CC genotypes were classified into low- and high-risk groups, respectively.

c KD patients with the GG or GA genotype and those with the AA genotypes were classified into low- and high-risk groups, respectively.

d KD patients with the GG genotype at one or both loci and those without the GG genotype at both loci were classified into low- and high-risk groups, respectively.

## Discussion

After the first case of KD was reported by Dr. Kawasaki in 1967 [36], KD has become the leading cause of acquired heart disease during childhood in developing countries in the past 40 years. Administration of a single dose of IVIG (2 g/kg) over a 12-hour period, combined with aspirin, is the standard treatment for KD. However, there are still 7.8–38% of KD patients who are unresponsive to initial IVIG treatment [5], [37], [38]. In this study, 43 (12.6%) KD patients didn’t respond well to initial IVIG treatment the incidence rate was compatible with other studies. In cases of persistent or recurrent inflammation after initial IVIG treatment, re-administration of IVIG or administration of other anti-inflammatory regimen should be considered [26], [39]. Early treatment of KD (5–10 days after disease onset) with IVIG results in better coronary outcomes and reduced total length of time of clinical symptoms [12], [40]. The purpose of this analysis is to help clinician identify children at higher risk of initial single-dose IVIG treatment failure. Kuo et al. [5], [26], reported that series of echocardiography examinations after IVIG treatment revealed that incidence of CAL formation was significantly higher in the IVIG-resistant group [5], [26].

The association between the functional polymorphism of *ITPKC* (rs28493229) and Kawasaki disease was firstly found by Onouchi et al. [20]. This SNP also contributed to the development of CAL formation. *ITPKC* (rs28493229) C allele with Kawasaki disease was further confirmed in Taiwanese population [19], [41]. Another functional genetic polymorphism of *CASP3*, rs113420705 (equally rs72689236), was reported to be associated with the susceptibility of Kawasaki disease by the same group of Japan and Taiwan [18], [21]. Interestingly, although susceptibility allele of *CASP3* showed a trend of correlation with Kawasaki disease in the Taiwanese population, *P*-value suggested a marginal association. Regarding to the genetic polymorphisms that involves into the risk of IVIG unresponsiveness, none of result was reported. By two locus association model, our results indicated that genotypes of *ITPKC* and *CASP3* contribute to a higher risk of IVIG resistance as well as CAL formation in the Taiwanese population. However, the results in the Taiwanese population are not as significant as that in the Japanese population. We attribute this to the different genetic backgrounds in two populations, due to variation in allele frequencies, population admixture, heterogeneity of the phenotype between populations.

CASP3, a key molecule in apoptosis pathways, has also been reported to cleave the inositol 1,4,5-triphosphate receptor, Type 1 (ITPR1) in T cells [42]. Compared to rs113420705 G allele of *CASP3*, risk allele A affects *CASP3* gene expression via change the binding of nuclear factor of activated T cells (NFAT) to the 5′ untranslated region (UTR) [21]. ITPKC, a negative regulator in Ca^2+^/NFAT pathways, controls immune responses in T cells. C allele rs28493229 of *ITPKC* may lose the ability to phosphorylate IP_3_. IP_3_ is a secondary message that triggers calcium release from cellular stores and activates calcium influx from ion channels. In non-excitable cells such as T cells, Ca^2+^ driven NFAT pathways regulate a variety of immune related genes. Thus, C allele rs28493229 of *ITPKC* may confer an aberrant of immune systems. The combinatorial effects of two risk alleles from two genes may cause more severe immune dysfunction and rigorous inflammatory reactions. In this case, additional anti-inflammation regiments after initial IVIG treatment should be prescribed earlier to minimize the cardiovascular sequel.

We recognize some potential limitations in this study. First, moderate sample size in this study may not have sufficient power to detect minor genetic effects. Second, only two polymorphisms were tested in this study which might not be able to fully reflect the genetic effects of CASP3 and ITPKC. In summary, we replicated the genetic effects of *CASP3* and *ITPKC* to CAL formation and IVIG resistance by a two locus model. A trend of overrepresentation of the IVIG non-responders was observed in the high-risk genotype group. This study might provide a clue for understanding the mechanism of IVIG unresponsiveness and CAL formation in the Kawasaki disease.

## Supporting Information

Figure S1 **We examined 12 combinatorial patterns in** ***ITPKC*** **(rs28493229) and** ***CAPS3*** **(rs113420705) 2-locus analysis.**
(TIF)
